# Sampling Inspection Plan to Test Daily COVID-19 Cases Using Gamma Distribution under Indeterminacy Based on Multiple Dependent Scheme

**DOI:** 10.3390/ijerph19095308

**Published:** 2022-04-27

**Authors:** Muhammad Aslam, Gadde Srinivasa Rao, Mohammed Albassam

**Affiliations:** 1 Department of Statistics, Faculty of Science, King Abdulaziz University, Jeddah 21551, Saudi Arabia; malbassam@kau.edu.sa; 2 Department of Mathematics and Statistics, University of Dodoma, Dodoma P.O. Box 259, Tanzania; gaddesrao@gmail.com

**Keywords:** COVID-19 data, multiple dependent state, single sampling plan, classical statistics, indeterminacy, average sample number, time-truncated sampling schemes

## Abstract

The purpose of this paper is to develop a multiple dependent state (MDS) sampling plan based on time-truncated sampling schemes for the daily number of cases of the coronavirus disease COVID-19 using gamma distribution under indeterminacy. The proposed sampling scheme parameters include average sample number (ASN) and accept and reject sample numbers when the indeterminacy parameter is known. In addition to the parameters of the proposed sampling schemes, the resultant tables are provided for different known indeterminacy parametric values. The outcomes resulting from various sampling schemes show that the ASN decreases as indeterminacy values increase. This shows that the indeterminacy parameter plays a vital role for the ASN. A comparative study between the proposed sampling schemes and existing sampling schemes based on indeterminacy is also discussed. The projected sampling scheme is illustrated with the help of the daily number of cases of COVID-19 data. From the results and real example, we conclude that the proposed MDS sampling scheme under indeterminacy requires a smaller sample size compared to the single sampling plan (SSP) and the existing MDS sampling plan.

## 1. Introduction

Nowadays, most of the countries in the world are affected by the current COVID-19 pandemic. COVID-19 is the infectious disease caused by the most recently discovered coronavirus. The most common symptoms of COVID-19 are fever, tiredness, and a dry cough. In more severe cases, the infection can cause pneumonia, severe acute respiratory syndrome, and even death. The number of cases in the pandemic is unknown in most countries worldwide. Aside from that, when cases are spreading, the usual practice of any country is to test the people who show more symptoms; on the other hand, there are some people that do not have any symptoms or only experience a few symptoms [1]. Through these less symptomatic people, the coronavirus spreads more in society. To identify these types of people, more health workers are employing the methodology of randomly testing chosen persons to approximately calculate the actual number of cases in a specified area and, hence, the total state. In such situations, an acceptance sampling plan under indeterminacy is a suitable alternative to testing or assessing the number of cases in a particular locality. Health workers endure pressure to approximate the average daily number of cases of COVID-19 at present and for the next few days, few weeks, or months. For more details, see [2]. The researchers or health workers are interested in testing the null hypothesis that the average daily number of cases is equivalent to the specified average daily number of cases of COVID-19 and the alternative hypothesis that the average daily number of cases of COVID-19 differs significantly. The null hypothesis may be rejected if the average daily number of cases of COVID-19 is more than or equal to the specified average daily number of cases of COVID-19 during the specified number of days.

Numerous authors have studied classical acceptance sampling plans based on time-condensed life tests using various life distributions. A few references to acceptance sampling plans can be seen in [3,4,5,6]. In recent years, different researchers have concentrated on a variety of sampling schemes, from single sampling plans (SSP) to multiple dependent state (MDS) sampling plans based on different distributions. The methodology of the MDS sampling plan was pioneered by [7], and it is based on the attribute assessment procedure, which is based on one out of three situations, namely, acceptance of the lot, rejection of the lot, or the conditional acceptance or rejection of the lot based on the character of future related lots. Subsequently, several authors studied MDS sampling designs on various distributions, such as [8,9,10,11,12,13,14,15,16,17,18].

Two decades ago, the author of [19] introduced a new perception of measurements, namely, neutrosophic logics, the measure of determinacy, and indeterminacy. Subsequently, various researchers discussed neutrosophic logic for a variety of valid problems and proved its effectiveness over fuzzy logic. For additional information, see [20,21,22,23,24,25]. The proposal of neutrosophic statistics was developed based on neutrosophic logic [26,27,28]. The author of [29] stated that neutrosophic statistics provide a lot of information on the measure of determinacy and the measure of indeterminacy. A generalization of traditional statistics is defined as neutrosophic statistics. For more information on acceptance sampling plans using neutrosophic statistics, refer to [30,31,32,33,34]. The authors of [35] proposed a single sampling plan for the inspection of COVID-19 patients using indeterminate Weibull distribution.

The aforementioned sampling designs using traditional statistics and a fuzzy environment do not provide background knowledge about the measure of indeterminacy. Reference [36] studied a single sampling plan based on a fuzzy environment. Reference [37] suggested the outcome of sampling error on assessment based on a fuzzy environment. Some other authors studied the single plan using a fuzzy logic environment; please refer to [38,39,40,41,42].

The present study is based on gamma distribution under indeterminacy. The gamma distribution is a generalization of exponential distribution and is associated with Erlang, normal, chi-square, beta, and some other distributions. This distribution was used for modeling in various life sciences such as epidemiology, computational biology, medical sciences, biostatistics, neuroscience, and so on. In recent years, more researchers studied the applications of gamma distribution in statistical quality control, reliability, queuing theory, survival analysis, and communication engineering. For more details, refer to [43]. The present research is motivated by the idea of neutrosophic statistics given by [26] and extensive studies by Aslam from 2018 onwards on various neutrosophic and indeterminacy probability distributions in different sampling and control chart schemes; some citations are given in the introduction section. Reference [44] originally introduced the indeterminate Weibull distribution and discussed its application in testing wind speed.

Having explored the current research literature related to sampling plans, we were able to conclude that our research work is pioneering, as there has been no previous research work on MDS sampling plan for gamma distribution under indeterminacy. In this paper, we introduce the MDS sampling plan for gamma distribution in the presence of uncertainty. The present piece of work is a targeted MDS sampling plan for gamma distribution under indeterminacy to test the daily number of cases occurring due to COVID-19. It is projected that the developed sampling design will demonstrate a smaller ASN compared with the on-hand sampling designs when testing the daily number of cases occurring due to COVID-19.

In Section 2, we provide a demonstration of the MDS sampling plan for gamma distribution (GD) under indeterminacy. Section 3 examines the comparative study with the existing sampling plans under indeterminacy, as well as existing, classical sampling plans. The proposed sampling plan for the indeterminacy is presented in Section 4 using a real example related to the daily number of cases occurring due to COVID-19. Section 5 deals with the conclusions and future research work.

## 2. Multiple Dependent State Sampling Plan under Indeterminacy

In this section, the development of the MDS sampling plan is discussed. The following are the essential conditions of pertinence to the proposed MDS sampling plan (see [16]):

(i) The inspection policy consists of taking successive lots manufactured from a continuous manufacturing process. This means that the lots are submitted for inspection serially in the order they have been manufactured in the manufacturing process;
(ii) The submitted lots for the examination have, for all intents and purposes, the same quality level. This means that the manufacturing process has a constant non-conforming fraction;
(iii) The consumer has assurance in the reliability of the manufacturer’s manufacturing process. It means that there is not any basis to consider that any specific lot quality is of inferior quality to the previous lots;
(iv) The quality attribute under contemplation follows a gamma distribution.

The MDS plan is an extension of the SSP. In the MDS plan, the lot-declaring scheme is developed from a one-critical-point to a two-critical-point plan, namely, a lot-accepted critical point $c_{1}$ and a lot-rejected critical point $c_{2}$, which allows the experiential quality level in between ($c_{2},c_{1}$) to judge the past, *m*-lot quality history. Based on this well-versed review, the MDS plan gains an advantage, economically, from governing smaller samples than the SSP.

The following, well-designed methodology for the MDS sampling design was given by [16] under neutrosophic statistics suggested by [44].

Step 1: Select a sample of size *n* from the batch. These specimens are employed for a life test for a predetermined time $t_{N0}$. Stipulate the average ${\;\mu }_{0N}$, and indeterminacy quantity is $I_{N}\;\epsilon \;\left[I_{L},I_{U}\right]$.

Step 2: The test $H_{0}:\mu_{N}=\mu_{0N}$ can be accepted if the average daily number of cases for $c_{1}$ days is greater or equal to ${\;\mu }_{0}$ (i.e.,$\;\mu_{0N}\leq c_{1}$). If the average daily number of cases in $c_{2}$ days is less than $\mu_{0}$ (i.e., $\mu_{0}$ *>* $c_{2}$), then test $H_{0}:\mu_{N}=\mu_{0N}$ can be rejected, and the test can be ended where $c_{1}\leq c_{2}$.

Step 3: When $c_{1}<\mu_{0N}\leq c_{2}$, then accept the current lot provided that, in *m* preceding lots, the mean number of cases is less than or equal to $c_{1}$ before the test termination time $t_{N0}$.

The planned MDS sampling plan under indeterminacy is totally differentiated by four values, namely, $n,c_{1},c_{2}$, and *m*, where *n* is the sample size, $c_{1}$ is the maximum number of allowable items that failed for unconditional acceptance, $c_{1}$, $c_{2}$ is the maximum number of additional items that failed for conditional acceptance $c_{1}\leq c_{2}$, and *m* is the number of successive lots (previous) needed to make a decision. The attributes’ MDS sampling plan converges to m→∞ and/or $c_{1}=c_{2}=c$ (say), and MDS is an oversimplification of SSP. The operating characteristic (OC) function can reveal the concert of any sampling design.

Applying binomial chance law, the OC function for the MDS sampling design based on GD is expressed as follows [16]:

(1) $$P_{a}\left(P_{N}\right)=P\left\{T\leq c_{1}\right\}+P\left\{c_{1}<T\leq c_{2}\right\}{\left[P\left\{T\leq c_{1}\right\}\right]}^{m}$$

where

$$P\left\{T\leq c_{1}\right\}=\sum_{d=0}^{c_{1}}\left(\begin{array}{l} n\\ d \end{array}\right)p_{N}^{d}{\left(1-p_{N}\right)}^{n-d}$$

and

$$P\left\{c_{1}<T\leq c_{2}\right\}=\sum_{d=c_{1}+1}^{c_{2}}\left(\begin{array}{l} n\\ d \end{array}\right)p_{N}^{d}{\left(1-p_{N}\right)}^{n-d}$$

$$P\left\{T\leq c_{2}\right\}=\sum_{d=0}^{c_{2}}\left(\begin{array}{l} n\\ d \end{array}\right)p_{N}^{d}{\left(1-p_{N}\right)}^{n-d}$$

where *T* is a random variable.

Thus, the final expression for the OC function for MDS sampling design is:

(2) $$P_{a}(p_{N})=\sum_{d=0}^{c_{1}}\left(\begin{array}{l} n\\ d \end{array}\right)\;p_{N}^{d}\;{\left(1-p_{N}\right)}^{\left(n-d\right)}+\sum_{d=c_{1}+1}^{c_{2}}\left(\begin{array}{l} n\\ d \end{array}\right)\;p_{N}^{d}\;{\left(1-p_{N}\right)}^{\left(n-d\right)}\times {\left[\sum_{d=0}^{c_{1}}\left(\begin{array}{l} n\\ d \end{array}\right)\;p_{N}^{d}\;{\left(1-p_{N}\right)}^{\left(n-d\right)}\right]}^{m}$$

The chance of lot approval is obtained at failure probability *p* under binomial probability distribution.

Suppose that $t_{N}\epsilon \left[t_{L},t_{U}\right]$ is the neutrosophic random variable that follows the gamma distribution. By following [44], let us assume that $f\left(t_{N}\right)=f\left(t_{L}\right)+f\left(t_{U}\right)I_{N};I_{N}\epsilon \left[I_{L},I_{U}\right]$ is a neutrosophic probability density function (npdf) with the determinate part $f\left(t_{L}\right)$, indeterminate part $f\left(t_{U}\right)I_{N}$, and indeterminacy period $I_{N}\epsilon \left[I_{L},I_{U}\right]$. Note that the measure of indeterminacy $I_{N}\epsilon \left[I_{L},I_{U}\right]$ presents uncertainty in the observations and parameters under an uncertain environment. Remember that $t_{N}\epsilon \left[t_{L},t_{U}\right]$ considers a neutrosophic random variable (nrv) which abides by the npdf. The npdf is the oversimplification of pdf based on traditional figures. Thus, the planned neutrosophic form of $f\left(t_{N}\right)\epsilon \left[f\left(t_{L}\right),f\left(t_{U}\right)\right]$ becomes the pdf of traditional figures as soon as $I_{L}$ = 0. Using this information, the npdf and neutrosophic cumulative distribution function (ncdf) of the GD is determined as under:

(3) $$\left(t_{N}\right)=\left\{\left(\theta^{\gamma }\Gamma \gamma \right)t_{N}^{\gamma -1}exp\left(-t_{N}/\theta \right)\right\}+\left\{\left(\theta^{\gamma }\Gamma \gamma \right)t_{N}^{\gamma -1}exp\left(-t_{N}/\theta \right)\right\}I_{N};\;I_{N}\epsilon \left[I_{L},I_{U}\right]$$

and

(4) $$F\left(t_{N}\right)=\left\{\frac{1}{\Gamma \gamma }\Gamma \left(\gamma ,t_{N}/\theta \right)\right\}+\left\{\frac{1}{\Gamma \gamma }\Gamma \left(\gamma ,t_{N}/\theta \right)\right\}I_{N};\;I_{N}\epsilon \left[I_{L},I_{U}\right].$$

where $\Gamma \left(\gamma ,t_{N}/\theta \right)$ is the lower incomplete gamma function, and (γ,θ). is the shape and scale parameters.

The average lifetime of the neutrosophic GD is $\mu_{0N}=\gamma \theta +\gamma \theta I_{N}$. A product failure probability before the time $t_{N0}$ is denoted as $p_{N}=F\left(t_{N}\leq t_{N0}\right)$ and is conveyed below:

(5) $$p_{N}=\left\{\frac{1}{\Gamma \gamma }\Gamma \left(\gamma ,t_{N}/\theta \right)\right\}+\left\{\frac{1}{\Gamma \gamma }\Gamma \left(\gamma ,t_{N}/\theta \right)\right\}I_{N}$$

Here, the neutrosophic termination time $t_{N0}$ is express as product of constant a  and neutrosophic mean life $\mu_{0N}$, i.e., $t_{N0}=a\mu_{0N}$. The scale parameter θ can be expressed in terms of the neutrosophic mean $\mu_{0N}$.

Therefore, Equation (4) can be rewritten in terms of the neutrosophic mean $\mu_{0N}$ as follows:

(6) $$p_{N}=\left\{\frac{1}{\Gamma \gamma }\Gamma \left(\gamma ,a\mu_{0N}/\theta \right)\right\}+\left\{\frac{1}{\Gamma \gamma }\Gamma \left(\gamma ,a\mu_{0N}/\theta \right)\right\}I_{N}\;=\left\{\frac{1}{\Gamma \gamma }\Gamma \left(\gamma ,\frac{a\mu_{0N}}{\mu_{N}/\theta }\right)\right\}+\left\{\frac{1}{\Gamma \gamma }\Gamma \left(\gamma ,\frac{a\mu_{0N}}{\mu_{N}/\theta }\right)\right\}I_{N}\;=\left\{\frac{1}{\Gamma \gamma }\Gamma \left(\gamma ,a\theta /\frac{\mu_{N}}{\mu_{0N}}\right)\right\}+\left\{\frac{1}{\Gamma \gamma }\Gamma \left(\gamma ,a\theta /\frac{\mu_{N}}{\mu_{0N}}\right)\right\}I_{N}$$

where $\mu_{N}/\mu_{0N}$ is the ratio of the exact average to the stipulated average.

Suppose that α and β are the probabilities of type-I and type-II errors. The researcher should pay attention to the projected plan when examining $H_{0}:\mu_{N}=\mu_{0N}$ in order to calculate the chance of accepting $H_{0}:\mu_{N}=\mu_{0N}$ as soon as the true quantity becomes at least 1−α at $\mu /\mu_{0}$ and the chance of accepting $H_{0}:\mu_{N}=\mu_{0N}$ as soon as the false quantity becomes at most β at $\mu_{N}/\mu_{0N}=1$. The plan constants for examining $H_{0}:\mu_{N}=\mu_{0N}$ are determined in such a way that the below two inequalities are fulfilled.

(7) $$L\left(p_{1N}|\mu_{N}/\mu_{0N}\right)\geq 1-\alpha$$

(8) $$L\left(p_{2N}|\mu_{N}/\mu_{0N}=1\right)\leq \beta$$

where $p_{1N}$ and $p_{2N}$ are defined by

(9) $$p_{1N}=\left\{\frac{1}{\Gamma \gamma }\Gamma \left(\gamma ,a\theta /\frac{\mu_{N}}{\mu_{0N}}\right)\right\}+\left\{\frac{1}{\Gamma \gamma }\Gamma \left(\gamma ,a\theta /\frac{\mu_{N}}{\mu_{0N}}\right)\right\}I_{N}$$

and

(10) $$p_{2N}=\left\{\frac{1}{\Gamma \gamma }\Gamma \left(\gamma ,a\theta \right)\right\}+\left\{\frac{1}{\Gamma \gamma }\Gamma \left(\gamma ,a\theta \right)\right\}I_{N}$$

Frequently, on-hand sampling schemes are intended to minimize the ASN. Commonly, the foremost intention of any sampling design is to decrease the ASN, which, in turn, minimizes both time and cost for inspection. Correspondingly, the projected MDS sampling design is intended to diminish the ASN for GD for the proposed situation. The non-linear programming method is adopted to get the optimal quantities, and it is expressed as follows:

(11) $$\mathrm{Minimize}\;ASN(p_{1N})=n\;\mathrm{Subject}\;\mathrm{to}\;P_{a}(p_{1N})\geq 1-\alpha \;P_{a}(p_{2N})\leq \beta \;n>1,m\geq 1,c_{2}>c_{1}\geq 0$$

where $p_{1N}$ and $p_{2N}$ are the failure probabilities of the producer’s and consumer’s risks, respectively. These acceptance chances can be found by means of the following expressions:

(12) $$P_{a}(p_{1N})=\sum_{d=0}^{c_{1}}\left(\begin{array}{l} n\\ d \end{array}\right)\;p_{1N}^{d}\;{\left(1-p_{1N}\right)}^{\left(n-d\right)}+\sum_{d=c_{1}+1}^{c_{2}}\left(\begin{array}{l} n\\ d \end{array}\right)\;p_{1N}^{d}\;{\left(1-p_{1N}\right)}^{\left(n-d\right)}\times {\left[\sum_{d=0}^{c_{1}}\left(\begin{array}{l} n\\ d \end{array}\right)\;p_{1N}^{d}{\left(1-p_{1N}\right)}^{\left(n-d\right)}\right]}^{m}$$

and

(13) $$P_{a}(p_{2N})=\sum_{d=0}^{c_{1}}\left(\begin{array}{l} n\\ d \end{array}\right)\;p_{2N}^{d}\;{\left(1-p_{2N}\right)}^{\left(n-d\right)}+\sum_{d=c_{1}+1}^{c_{2}}\left(\begin{array}{l} n\\ d \end{array}\right)\;p_{2N}^{d}\;{\left(1-p_{2N}\right)}^{\left(n-d\right)}\times {\left[\sum_{d=0}^{c_{1}}\left(\begin{array}{l} n\\ d \end{array}\right)\;p_{2N}^{d}{\left(1-p_{2N}\right)}^{\left(n-d\right)}\right]}^{m}$$

The proposed plan consists of parameters $c_{1},c_{2},m\;\mathrm{and}\;ASN$ is obtained by solving the non-linear programming problem in Equation (11) for β={0.25,0.10,0.05}, α=0.10, and a=0.5, 1.0, and known $I_{N}$ is placed in Table 1, Table 2, Table 3, Table 4, Table 5, Table 6, Table 7 and Table 8. Table 1 and Table 2 show the GD for γ=2, Table 3 and Table 4 show the GD for γ=2.5, Table 5 and Table 6 for γ=3, Table 5 and Table 6 for γ=3, and Table 7 and Table 8 for γ=1. (exponential distribution). From the results in the tables, the following few points can be noticed:

(a) The values of the ASN decrease as the value of a increases from 0.5 to 1.0;
(b) The ASN decreases as the shape parameter increases from γ=1 to θ=3  when other parameters are fixed;
(c) In addition, to minimize ASN values, the indeterminacy parameter $I_{N}$. shows a significant influence;
(d) The ASN is larger for the traditional MDS plan than the proposed MDS sampling plan under indeterminacy;
(e) From Table 7 and Table 8, it can be observed that GD shows a small ASN compared with exponential distribution;
(f) Furthermore, it is depicted by the OC curves that the proposed MDS sampling plan under indeterminacy is more efficient than the existing single sampling plan.

## 3. Comparative Studies

**Figure 1:**
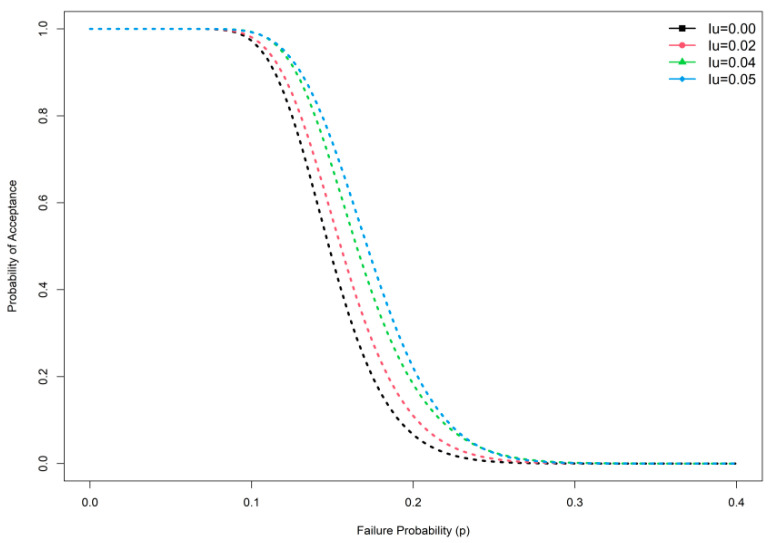
OC curve plan at different indeterminacy values.

**Figure 2:**
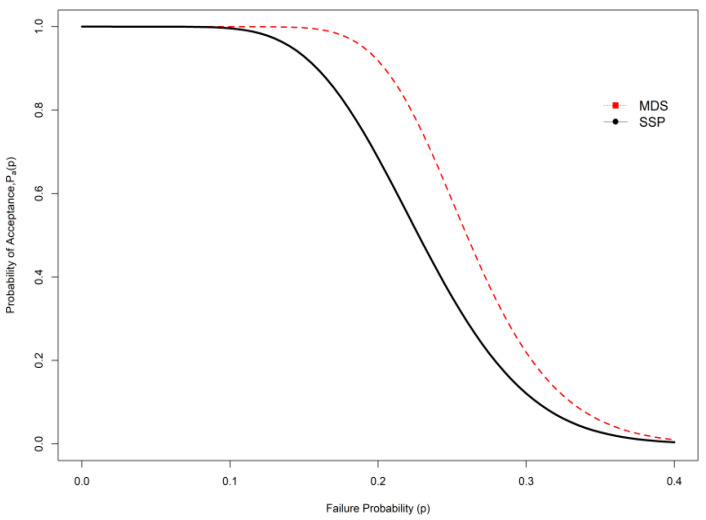
OC curve comparison between SSP and MDS under indeterminacy.

This section deals with a comparative study of the proposed sampling scheme with the existing SSP. The efficiency of the developed sampling plan is calculated based on the ASN; a low-sample-size design is more economical to test the hypothesis about the mean. It is important to note that the proposed MDS sampling plan under indeterminacy is the generalization of the MDS sampling plan for traditional statistics if no uncertainty or indeterminacy happens when measuring the average. If $I_{N}$ = 0, the proposed MDS sampling plan under indeterminacy becomes the MDS sampling plan in hand. In Table 1, Table 2, Table 3, Table 4, Table 5, Table 6, Table 7 andTable 8, the first column, i.e., at $I_{N}$ = 0, is the plan parameter of the traditional or existing MDS sampling plan. From the results, we conclude that the ASN is large in the traditional MDS sampling plan compared with the proposed MDS sampling plan. For example, when α=0.10,β=0.25, $\mu_{N}/\mu_{0N}$ = 1.4, γ = 2, and a = 0.5, from Table 1 it can be seen that ASN = 39 from the plan under classical statistics, and ASN = 34 for the projected sampling plan when $I_{N}$ = 0.05. Furthermore, when γ = 1, the GD becomes an exponential distribution; we constructed Table 7 and Table 8 for exponential distribution for comparison purposes. Table 7 depicts that the GD shows a lower sample number compared with exponential distribution. For example, when α=0.10,β=0.25, $\mu_{N}/\mu_{0N}$ = 1.5, a = 0.5, and $I_{N}$ = 0.04, Table 7 shows that the ASN is 42, whereas the proposed plan values are *ASN* = 25 for γ = 2, *ASN* = 24 for γ = 2.5, and *ASN* = 20 for γ = 3. From this study, it is concluded that the projected plan under indeterminacy is more efficient than the existing sampling plan under traditional statistics with respect to sample size. The operating characteristic (OC) curve of the plan of the GD when α=0.10,β=0.10, γ=3.0, and  a=0.50 is depicted in Figure 1 and Figure 2. Therefore, the application of the proposed plan for testing the null hypothesis $H_{0}:\mu_{N}=\mu_{0N}$ demands a smaller sample compared to the on-hand plan. The OC curve in Figure 1 also shows the same performance. Researchers can apply the proposed plan under uncertainty to save time and money.

## 4. Applications for COVID-19 Data

The practical utility of the anticipated sampling design for the GD under indeterminacy is presented in this section using a real example. The real data set is constituted of newly notified cases on a daily basis. These data consist of a 61-day COVID-19 data set taken from Italy, which reports the daily number of cases between 13 June and 12 August 2021. For more details, refer to the new, discrete distribution with application to COVID-19 data developed by [45]. For ready reference, the data are reported here:

Daily number of cases: 52, 26, 36, 63, 52, 37, 35, 28, 17, 21, 31, 30, 10, 56, 40, 14, 28, 42, 24, 21, 28, 22, 12, 31, 24, 14, 13, 25, 12, 7, 13, 20, 23, 9, 11, 13, 3, 7, 10, 21, 15, 17, 5, 7, 22, 24, 15, 19, 18, 16, 5, 20, 27, 21, 27, 24, 22, 11, 22, 31, and 31.

The daily number of cases occurring due to COVID-19 plays a vital role for medical administrators in every nation in the world nowadays. COVID-19 cases encounter unpredictability and uncertainty; thus, the daily number of cases data become a probability distribution under neutrosophic information. The government administrators are anxious to monitor the average daily number of cases under indeterminacy.

**Figure 3:**
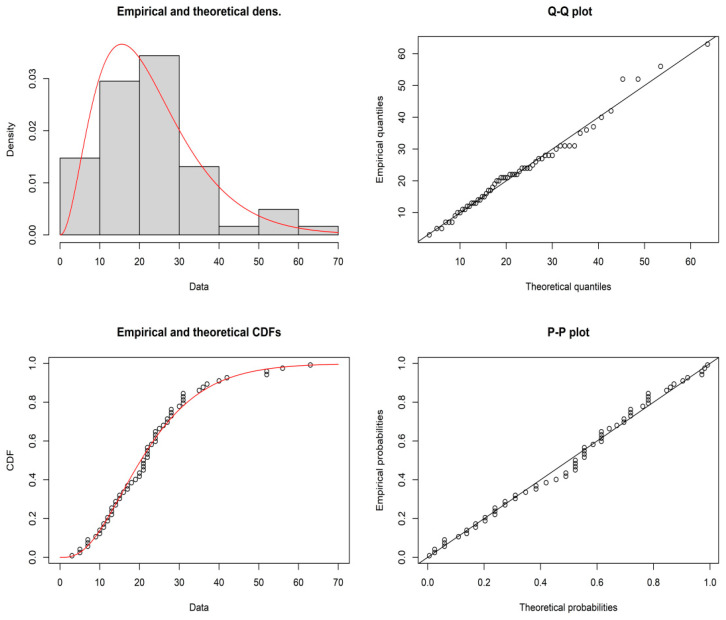
Pictorial presentation of various plots for the GD for daily number of cases data.

It is established that the daily number of cases data can be drawn from the GD with shape parameter $\hat{\gamma }$ = 3.1946 and scale parameter $\hat{\theta }=7.0809$, and the maximum distance between the real-time data and the fitted of GD can be found from the Kolmogorov–Smirnov test statistic 0.0803 and also the *p*-value 0.8262. The demonstration of the goodness of fit for the given model, the empirical and theoretical pdfs, cdf, P-P plot and Q-Q plots for the GD for the daily number of cases data are shown in Figure 3. Therefore, GD is well fitted for the daily number of cases of COVID-19 data. The plan parameters for this shape parameter are shown in Table 9 and Table 10. For the proposed plan, the shape parameter is ${\hat{\gamma }}_{N}=\left(1+0.04\right)\times 3.1946\approx 3.3224$ when $I_{U}$ = 0.04. Suppose that medical administrators are concerned with testing $H_{0}:\mu_{N}=23.5255$ with the aid of the proposed sampling plan when $I_{U}$ = 0.04, α=0.10, $\mu_{N}/\mu_{0N}$ = 1.4, a = 0.5, and β = 0.10. From Table 9, it can be noted that n = 55, $c_{1}=6$, $c_{2}$ = 14, *m* = 1, and *ASN* = 55. The developed MDS sampling plan works by accepting the null hypothesis $H_{0}:\mu_{N}=23.5255$ if the average daily number of cases in 6 days is more than equal to 23.5255 daily number of cases. A sample of 55 daily due to COVID-19 can be selected at random for a crowd of people, and the null hypothesis ${\;H}_{0}:\mu_{N}=23.5255$. If the average daily number of cases before 23.5255 is less than or equal to 6 days, then the crowd of people can be accepted, and the crowd of people can be rejected if it is greater than 14 days. If the prevalence of the number of cases of COVID-19 is between 6 and 14 days, a property of the present crowd of people can be deferred until the preceding crowd of people has be tested. From the data, it can be noted that an average daily number of cases of COVID-19 was greater than equal to 23.5255 encounter in more than 36 days; therefore, the claim about the average daily number of cases $H_{0}:\mu_{N}=23.5255$ could be rejected. Hence, medical administrators could suggest to the government that the average daily number of cases of COVID-19 was at an unendurable stage. Therefore, the proposed sampling plan is useful in medical applications, specifically, in taking decisions regarding the daily number of cases of COVID-19 and the average daily number of COVID-19 patients, and this is very important for any government making policy decisions.

## 5. Conclusions

A broad analysis of the daily number of cases of COVID-19 for gamma distribution based on indeterminacy situation for a time-truncated MDS sampling design was formulated. The sampling plan’s quantities were determined at pre-assigned values of indeterminacy parameters. Comprehensive tables were given for ready reference to the researchers for the known indeterminacy constant values. The formulated MDS sampling design based on indeterminacy was compared with the already available sampling schemes based on classical statistics. The results showed that the formulated MDS sampling plan under indeterminacy was more reasonable than the already available SSP under indeterminacy and traditional MDS sampling plans. In addition, the developed MDS under indeterminacy was greatly cheaper to run than the SSP. It is important to note that the indeterminacy parameter showed a prime role in decreasing ASN values, which means that, if the indeterminacy value increased then the ASN values were in decreasing trend. Therefore, the formulated MDS sampling plan under indeterminacy is more useful to scientists, in particular to medical practitioners and those who are studying or testing sensitive issues which require skilled researchers and need more money. Thus, the formulated MDS sampling plan under indeterminacy is approved to be applicable for testing the average daily number of cases of COVID-19. The exemplification based on the daily number of cases of COVID-19 data for the formulated MDS sampling plan under indeterminacy showed confirmation. The formulated MDS sampling plan under indeterminacy can be used by other researchers working in various fields. Considering a control chart methodology based on multiple dependent state sampling plans will be the topic of a further research study to monitor the mean.

## Figures and Tables

**Table 1:** The MDS design values for α=0.10, ν=2.0, and a=0.5.

β	$\frac{\mu_{N}}{\mu_{0N}}$	$I_{U}=0.00$					$I_{U}=0.02$					$I_{U}=0.04$					$I_{U}=0.05$				
		$c_{1}$	$c_{2}$	m	$L\left(p_{1}\right)$	*ASN*	$c_{1}$	$c_{2}$	m	$L\left(p_{1}\right)$	*ASN*	$c_{1}$	$c_{2}$	m	$L\left(p_{1}\right)$	*ASN*	$c_{1}$	$c_{2}$	m	$L\left(p_{1}\right)$	*ASN*
0.25	1.2	25	32	2	0.9039	110	26	33	2	0.9059	109	26	33	2	0.9058	104	26	74	2	0.9072	102
	1.3	12	16	2	0.9038	57	12	31	2	0.9053	55	12	18	2	0.9100	52	12	17	2	0.9033	51
	1.4	7	30	2	0.9021	39	7	30	2	0.9061	35	7	11	1	0.9141	35	8	33	4	0.9076	34
	1.5	5	16	2	0.9220	28	5	19	3	0.9088	26	5	15	3	0.9035	25	5	13	3	0.9129	24
	1.8	2	9	2	0.9185	15	2	12	2	0.9239	14	2	6	3	0.9059	13	2	10	2	0.9236	13
	2.0	1	4	2	0.9086	10	1	3	1	0.9093	11	1	5	2	0.9095	9	1	9	2	0.9027	9
0.1	1.2	44	52	1	0.9008	203	44	53	1	0.9020	194	44	52	1	0.9051	184	45	60	2	0.9002	181
	1.3	20	28	1	0.9033	103	21	69	2	0.9016	99	22	73	2	0.9140	98	21	68	2	0.9037	92
	1.4	12	17	1	0.9002	68	12	18	1	0.9036	65	12	19	1	0.9062	62	12	16	1	0.9052	59
	1.5	8	14	2	0.9050	47	8	14	2	0.9002	45	8	11	1	0.9023	44	8	12	1	0.9030	44
	1.8	3	7	1	0.9043	26	3	6	1	0.9080	24	3	6	1	0.9047	23	3	8	1	0.9047	23
	2.0	3	7	3	0.9300	24	2	4	1	0.9067	19	2	7	1	0.9125	19	2	5	1	0.9162	18
0.05	1.2	58	67	1	0.9025	269	58	68	1	0.9030	257	58	68	1	0.9038	245	59	68	1	0.9022	243
	1.3	27	37	1	0.9050	140	28	38	1	0.9163	137	26	34	1	0.9013	122	28	37	2	0.9026	124
	1.4	17	80	2	0.9054	93	16	24	1	0.9091	87	16	23	1	0.9045	83	16	23	1	0.9049	81
	1.5	10	18	1	0.9002	64	10	15	1	0.9006	60	10	15	1	0.9023	57	10	14	1	0.9002	55
	1.8	4	8	1	0.9043	34	5	17	2	0.9154	36	5	7	1	0.9207	34	5	29	2	0.9212	33
	2.0	3	6	2	0.9066	27	3	17	2	0.9058	26	3	30	2	0.9006	25	3	17	2	0.9073	24

**Table 2:** The MDS design values for α=0.10,ν=2.0, and a=1.0.

β	$\frac{\mu_{N}}{\mu_{0N}}$	$I_{U}=0.00$					$I_{U}=0.02$					$I_{U}=0.04$					$I_{U}=0.05$				
		$c_{1}$	$c_{2}$	m	$L\left(p_{1}\right)$	*ASN*	$c_{1}$	$c_{2}$	m	$L\left(p_{1}\right)$	*ASN*	$c_{1}$	$c_{2}$	m	$L\left(p_{1}\right)$	*ASN*	$c_{1}$	$c_{2}$	m	$L\left(p_{1}\right)$	*ASN*
0.25	1.2	34	38	2	0.9013	63	33	39	2	0.9104	59	33	53	2	0.9101	57	32	36	1	0.9001	55
	1.3	15	21	1	0.9037	31	16	19	1	0.9017	31	15	18	1	0.9019	28	14	18	1	0.9030	26
	1.4	9	18	2	0.9042	19	9	12	2	0.9083	18	9	12	3	0.9021	17	9	12	2	0.9088	17
	1.5	7	9	3	0.9058	15	6	11	2	0.9033	13	6	12	3	0.9065	12	6	9	2	0.9186	12
	1.8	3	6	2	0.9257	8	2	4	1	0.9041	6	3	5	4	0.9066	7	3	6	2	0.9371	7
	2.0	2	13	2	0.9330	6	2	5	2	0.9179	6	2	6	1	0.9435	6	2	5	5	0.9081	5
0.1	1.2	59	75	2	0.9063	112	58	83	2	0.9020	106	58	66	1	0.9175	103	54	64	1	0.9006	95
	1.3	26	32	1	0.9006	54	25	32	1	0.9017	50	26	34	1	0.9047	50	25	31	1	0.9030	47
	1.4	16	20	1	0.9159	35	15	29	2	0.9015	31	15	21	1	0.9039	31	15	19	2	0.9037	29
	1.5	11	19	2	0.9148	25	10	18	2	0.9029	22	10	14	2	0.9052	21	12	16	3	0.9162	24
	1.8	4	7	1	0.9085	12	5	8	2	0.9185	13	4	7	1	0.9090	11	3	6	2	0.9142	11
	2.0	3	5	1	0.9141	10	3	6	2	0.9141	9	3	6	1	0.9359	9	3	6	1	0.9279	9
0.05	1.2	74	82	1	0.9035	143	73	82	1	0.9024	136	73	84	1	0.9075	131	72	85	1	0.9056	127
	1.3	35	43	1	0.9076	73	35	42	1	0.9060	70	33	38	1	0.9018	63	33	39	1	0.9067	62
	1.4	20	27	1	0.9051	45	20	26	1	0.9067	43	20	26	1	0.9166	41	20	24	1	0.9052	40
	1.5	13	17	1	0.9070	31	14	26	2	0.9087	31	13	19	1	0.9041	29	13	17	1	0.9075	28
	1.8	7	12	3	0.9060	19	6	10	2	0.9091	16	6	10	1	0.9269	16	6	11	2	0.9111	15
	2.0	4	13	1	0.9092	14	4	7	2	0.9117	12	4	7	1	0.9315	12	4	6	2	0.9129	11

**Table 3:** The MDS design values for α=0.10,ν=2.5, and a=0.50.

β	$\frac{\mu_{N}}{\mu_{0N}}$	$I_{U}=0.00$					$I_{U}=0.02$					$I_{U}=0.04$					$I_{U}=0.05$				
		$c_{1}$	$c_{2}$	m	$L\left(p_{1}\right)$	*ASN*	$c_{1}$	$c_{2}$	m	$L\left(p_{1}\right)$	*ASN*	$c_{1}$	$c_{2}$	m	$L\left(p_{1}\right)$	*ASN*	$c_{1}$	$c_{2}$	m	$L\left(p_{1}\right)$	*ASN*
0.25	1.2	18	24	2	0.9060	97	18	25	1	0.9003	97	18	47	2	0.9015	88	18	24	2	0.9042	85
	1.3	8	13	1	0.9010	52	8	31	2	0.9015	46	9	32	3	0.9084	47	8	13	1	0.9065	45
	1.4	5	23	2	0.9069	34	5	17	2	0.9091	32	5	27	3	0.9051	29	5	9	2	0.9147	29
	1.5	3	10	2	0.9088	23	3	66	2	0.9034	22	4	12	3	0.9247	24	3	11	2	0.9085	20
	1.8	1	8	2	0.9113	12	1	14	1	0.9070	14	1	6	2	0.9032	11	1	6	1	0.9034	13
	2.0	1	3	4	0.9179	12	1	9	4	0.9260	11	1	8	4	0.9226	10	1	5	3	0.9417	10
0.1	1.2	31	38	1	0.9004	177	32	65	2	0.9016	169	32	78	2	0.9033	160	32	72	2	0.9019	156
	1.3	15	38	2	0.9086	93	15	67	2	0.9081	88	14	19	1	0.9026	81	15	22	2	0.9075	81
	1.4	8	14	1	0.9020	60	8	16	1	0.9012	57	8	13	1	0.9055	53	8	12	1	0.9039	51
	1.5	5	9	1	0.9076	42	6	23	2	0.9215	43	5	9	1	0.9005	38	5	8	1	0.9016	36
	1.8	2	6	1	0.9178	25	2	5	1	0.9204	23	2	5	2	0.9015	20	3	15	5	0.9024	25
	2.0	2	19	2	0.9546	23	2	12	6	0.9100	21	2	7	5	0.9168	20	1	4	1	0.9027	16
0.05	1.2	40	49	1	0.9002	231	41	56	1	0.9010	226	41	54	1	0.9006	214	43	71	2	0.9015	212
	1.3	20	29	2	0.9049	126	19	25	1	0.9048	117	20	68	2	0.9046	113	20	51	2	0.9048	110
	1.4	11	15	1	0.9056	80	11	18	1	0.9040	78	11	15	1	0.9009	72	11	16	1	0.9026	71
	1.5	7	13	1	0.9014	60	7	10	1	0.9022	54	7	10	1	0.9031	51	7	12	1	0.9106	51
	1.8	3	13	2	0.9073	33	3	7	2	0.9073	31	3	17	2	0.9122	29	3	18	2	0.9003	29
	2.0	2	4	2	0.9148	26	2	16	2	0.9200	25	2	18	2	0.9005	25	2	17	2	0.9186	23

**Table 4:** The MDS design values for α=0.10,ν=2.5, and a=1.00.

β	$\frac{\mu_{N}}{\mu_{0N}}$	$I_{U}=0.00$					$I_{U}=0.02$					$I_{U}=0.04$					$I_{U}=0.05$				
		$c_{1}$	$c_{2}$	m	$L\left(p_{1}\right)$	*ASN*	$c_{1}$	$c_{2}$	m	$L\left(p_{1}\right)$	*ASN*	$c_{1}$	$c_{2}$	m	$L\left(p_{1}\right)$	*ASN*	$c_{1}$	$c_{2}$	m	$L\left(p_{1}\right)$	*ASN*
0.25	1.2	26	30	2	0.9007	50	25	29	2	0.9041	46	26	46	2	0.9187	46	25	33	3	0.9039	43
	1.3	12	24	2	0.9040	25	11	16	1	0.9024	23	11	16	2	0.9034	21	12	16	3	0.9015	22
	1.4	7	9	1	0.9027	16	7	11	2	0.9068	15	7	12	3	0.9019	14	7	13	2	0.9123	14
	1.5	5	7	2	0.9011	12	5	8	3	0.9175	11	5	7	2	0.9157	11	5	9	2	0.9013	11
	1.8	2	5	2	0.9312	6	2	3	2	0.9144	6	2	5	1	0.9395	6	2	4	4	0.9151	5
	2.0	1	4	2	0.9154	4	1	2	2	0.9004	4	2	4	3	0.9299	6	1	2	1	0.9274	4
0.1	1.2	46	54	1	0.9161	92	42	50	1	0.9021	81	42	53	1	0.9004	78	42	50	1	0.9067	76
	1.3	20	27	1	0.9039	44	20	26	1	0.9035	42	20	82	2	0.9007	39	19	23	1	0.9019	37
	1.4	12	15	1	0.9058	28	11	15	1	0.9056	25	12	18	2	0.9113	25	13	21	3	0.9092	26
	1.5	8	10	1	0.9004	20	8	16	2	0.9020	19	8	14	2	0.9099	18	9	13	3	0.9231	19
	1.8	4	9	2	0.9334	12	3	8	2	0.9097	9	4	7	2	0.9295	11	3	6	1	0.9218	9
	2.0	2	4	1	0.9214	8	2	5	2	0.9179	7	3	8	2	0.9516	9	3	5	3	0.9238	9
0.05	1.2	59	67	1	0.9024	119	60	69	2	0.9039	114	57	81	2	0.9027	104	57	72	2	0.9004	102
	1.3	26	32	1	0.9068	57	26	35	1	0.9044	55	26	31	1	0.9065	52	25	30	1	0.9057	49
	1.4	15	20	1	0.9037	36	15	19	1	0.9067	34	15	21	1	0.9067	33	15	21	1	0.9168	32
	1.5	11	15	2	0.9180	27	11	14	2	0.9014	26	10	15	1	0.9065	24	11	19	2	0.9282	24
	1.8	4	12	1	0.9062	14	4	8	1	0.9162	13	5	6	2	0.9245	14	4	7	1	0.9143	12
	2.0	3	5	2	0.9162	11	3	7	3	0.9096	10	3	5	2	0.9139	10	3	5	2	0.9026	10

**Table 5:** The MDS design values for α=0.10,ν=3, and a=0.50.

β	$\frac{\mu_{N}}{\mu_{0N}}$	$I_{U}=0.00$					$I_{U}=0.02$					$I_{U}=0.04$					$I_{U}=0.05$				
		$c_{1}$	$c_{2}$	m	$L\left(p_{1}\right)$	*ASN*	$c_{1}$	$c_{2}$	m	$L\left(p_{1}\right)$	*ASN*	$c_{1}$	$c_{2}$	m	$L\left(p_{1}\right)$	*ASN*	$c_{1}$	$c_{2}$	m	$L\left(p_{1}\right)$	*ASN*
0.25	1.2	13	49	2	0.9016	86	13	74	2	0.9005	81	13	19	2	0.9005	76	13	20	2	0.9004	74
	1.3	6	10	1	0.9125	48	6	9	2	0.9003	42	6	10	2	0.9017	40	6	20	2	0.9041	39
	1.4	3	7	1	0.9005	30	4	21	4	0.9051	30	4	12	3	0.9102	29	4	17	3	0.9125	28
	1.5	2	11	2	0.9022	21	2	15	1	0.9008	23	2	4	1	0.9027	20	2	9	2	0.9007	18
	1.8	1	10	4	0.9156	14	1	11	4	0.9174	13	1	8	4	0.9209	12	1	6	3	0.9295	12
	2.0	1	8	7	0.9403	14	1	7	23	0.9010	13	1	4	4	0.9628	12	1	5	6	0.9017	12
0.1	1.2	24	50	2	0.9028	163	24	42	2	0.9053	153	24	31	1	0.9040	149	25	32	2	0.9072	145
	1.3	10	15	1	0.9004	82	11	20	2	0.9065	80	11	17	1	0.9139	79	11	17	2	0.9047	73
	1.4	6	10	1	0.9070	57	6	15	1	0.9035	55	6	9	1	0.9069	49	6	22	2	0.9006	46
	1.5	4	9	2	0.9061	41	2	15	1	0.9008	23	4	20	2	0.9074	36	4	17	2	0.9062	35
	1.8	2	6	4	0.9146	27	2	13	5	0.9049	25	2	11	2	0.9468	24	2	13	4	0.9137	23
	2.0	1	6	3	0.9204	19	1	12	3	0.9172	18	1	9	3	0.9151	17	1	11	3	0.9069	17
0.05	1.2	32	81	2	0.9026	220	32	83	2	0.9022	207	31	42	1	0.9069	195	25	32	2	0.9072	145
	1.3	15	68	2	0.9056	118	15	68	2	0.9044	111	15	52	2	0.9084	104	14	22	1	0.9021	100
	1.4	8	18	1	0.9043	78	8	14	1	0.9111	72	8	12	1	0.9033	67	8	15	1	0.9098	66
	1.5	5	19	1	0.9142	55	5	13	1	0.9002	54	5	10	1	0.9141	49	5	11	1	0.9006	49
	1.8	2	14	2	0.9141	31	2	4	2	0.9012	29	2	17	2	0.9040	28	2	16	2	0.9055	27
	2.0	1	3	1	0.9151	25	1	5	1	0.9215	24	1	15	1	0.9060	24	1	4	1	0.9166	22

**Table 6:** The MDS design values for α=0.10,ν=3, and a=1.0.

β	$\frac{\mu_{N}}{\mu_{0N}}$	$I_{U}=0.00$					$I_{U}=0.02$					$I_{U}=0.04$					$I_{U}=0.05$				
		$c_{1}$	$c_{2}$	m	$L\left(p_{1}\right)$	*ASN*	$c_{1}$	$c_{2}$	m	$L\left(p_{1}\right)$	*ASN*	$c_{1}$	$c_{2}$	m	$L\left(p_{1}\right)$	*ASN*	$c_{1}$	$c_{2}$	m	$L\left(p_{1}\right)$	*ASN*
0.25	1.2	20	36	2	0.9011	40	20	25	2	0.9050	38	21	25	2	0.9091	38	19	31	2	0.9004	34
	1.3	10	24	3	0.9114	21	10	17	3	0.9141	20	10	14	4	0.9019	19	9	17	3	0.9005	17
	1.4	6	11	2	0.9208	14	6	11	3	0.9133	13	6	11	2	0.9067	13	6	9	4	0.9044	12
	1.5	4	9	2	0.9326	10	4	10	2	0.9073	10	4	8	3	0.9136	9	4	8	2	0.9257	9
	1.8	1	3	2	0.9025	4	2	5	4	0.9146	6	2	4	2	0.9358	6	2	4	3	0.9021	6
	2.0	1	2	3	0.9292	4	1	4	3	0.9152	4	1	2	2	0.9255	4	1	2	2	0.9186	4
0.1	1.2	36	44	2	0.9070	73	35	52	2	0.9025	68	35	52	2	0.9044	65	34	40	1	0.9042	63
	1.3	17	20	1	0.9035	38	16	22	1	0.9101	35	17	31	2	0.9176	34	16	19	1	0.9023	32
	1.4	10	19	2	0.9150	24	9	14	1	0.9027	22	9	15	1	0.9041	21	10	17	3	0.9020	21
	1.5	6	12	2	0.9028	16	6	11	2	0.9125	15	6	9	1	0.9168	15	6	9	2	0.9105	14
	1.8	3	8	3	0.9126	10	2	7	1	0.9004	8	3	4	3	0.9125	9	2	4	1	0.9068	7
	2.0	2	5	3	0.9160	8	2	4	3	0.9402	7	2	3	4	0.9069	7	2	3	2	0.9376	7
0.05	1.2	46	54	1	0.9063	96	47	76	2	0.9042	92	46	52	1	0.9095	87	45	54	1	0.9089	84
	1.3	21	26	1	0.9093	48	20	26	1	0.9050	44	20	26	1	0.9057	42	21	27	1	0.9099	43
	1.4	12	15	1	0.9005	30	12	22	2	0.9021	28	12	20	1	0.9061	28	12	20	2	0.9058	26
	1.5	8	19	1	0.9034	23	8	11	1	0.9118	21	8	12	1	0.9245	20	8	17	2	0.9037	19
	1.8	3	9	1	0.9086	12	3	7	1	0.9207	11	4	9	3	0.9171	12	4	10	3	0.9034	12
	2.0	2	4	1	0.9307	9	2	4	1	0.9277	9	3	4	3	0.9071	9	3	5	5	0.9068	9

**Table 7:** The MDS design values for α=0.10,ν=1, and a=0.50.

β	$\frac{\mu_{N}}{\mu_{0N}}$	$I_{U}=0.00$					$I_{U}=0.02$					$I_{U}=0.04$					$I_{U}=0.05$				
		$c_{1}$	$c_{2}$	m	$L\left(p_{1}\right)$	*ASN*	$c_{1}$	$c_{2}$	m	$L\left(p_{1}\right)$	*ASN*	$c_{1}$	$c_{2}$	m	$L\left(p_{1}\right)$	*ASN*	$c_{1}$	$c_{2}$	m	$L\left(p_{1}\right)$	*ASN*
0.25	1.2	74	85	2	0.9127	203	74	84	2	0.9107	196	71	83	2	0.9088	182	71	82	2	0.9088	181
	1.3	35	46	3	0.9022	99	35	61	2	0.9061	97	35	51	2	0.9010	94	33	52	2	0.9004	87
	1.4	21	60	2	0.9014	63	22	60	3	0.9114	62	22	25	2	0.9026	60	22	40	3	0.9094	59
	1.5	15	32	2	0.9010	47	15	44	3	0.9056	44	11	16	4	0.9002	42	14	35	3	0.9020	39
	1.8	7	23	2	0.9227	24	7	20	3	0.9009	23	7	17	3	0.9080	22	6	20	1	0.9015	21
	2.0	5	13	3	0.9109	18	5	12	4	0.9053	17	5	9	2	0.9284	16	4	13	2	0.9013	14
0.1	1.2	-	-	-	-	-	-	-	-	-	-	-	-	-	-	-	-	-	-	-	-
	1.3	63	80	2	0.9099	188	66	82	2	0.9245	185	61	70	2	0.9012	166	58	71	1	0.9040	159
	1.4	40	85	2	0.9129	122	38	71	2	0.9077	112	37	82	1	0.9018	109	35	47	2	0.9016	98
	1.5	27	58	2	0.9248	85	25	61	2	0.9029	77	25	68	2	0.9096	74	24	42	2	0.9042	70
	1.8	12	41	2	0.9091	48	13	34	3	0.9048	44	12	36	3	0.9058	39	12	24	2	0.9171	37
	2.0	8	26	2	0.9045	33	8	24	2	0.9019	30	8	21	2	0.9007	29	8	21	1	0.9150	27
0.05	1.2	-	-	-	-	-	-	-	-	-	-	-	-	-	-	-	-	-	-	-	-
	1.3	-	-	-	-	-	-	-	-	-	-	-	-	-	-	-	-	-	-	-	-
	1.4	47	59	1	0.9015	149	48	78	2	0.9024	143	48	69	1	0.9025	142	49	81	2	0.9119	138
	1.5	35	57	2	0.9045	113	34	71	2	0.9016	106	35	60	2	0.9089	105	33	59	1	0.9003	101
	1.8	16	47	2	0.9105	58	16	41	1	0.9078	57	16	49	2	0.9106	54	16	41	2	0.9119	53
	2.0	11	20	1	0.9205	45	11	19	2	0.9172	41	10	30	1	0.9008	39	11	33	2	0.9150	37

(-) represents parameters do not exist.

**Table 8:** The MDS design values for α=0.10,ν=1, and a=1.0.

β	$\frac{\mu_{N}}{\mu_{0N}}$	$I_{U}=0.00$					$I_{U}=0.02$					$I_{U}=0.04$					$I_{U}=0.05$				
		$c_{1}$	$c_{2}$	m	$L\left(p_{1}\right)$	*ASN*	$c_{1}$	$c_{2}$	m	$L\left(p_{1}\right)$	*ASN*	$c_{1}$	$c_{2}$	m	$L\left(p_{1}\right)$	*ASN*	$c_{1}$	$c_{2}$	m	$L\left(p_{1}\right)$	*ASN*
0.25	1.2	75	84	2	0.9072	126	77	84	2	0.9116	125	76	83	1	0.9133	121	76	82	1	0.9133	119
	1.3	38	54	2	0.9110	68	39	58	2	0.9007	66	36	45	3	0.9089	58	37	52	3	0.9007	56
	1.4	25	35	4	0.9016	44	23	32	2	0.9100	40	23	29	3	0.9170	38	21	28	2	0.9003	35
	1.5	14	24	2	0.9031	29	15	24	2	0.9054	27	15	25	2	0.9143	26	15	18	4	0.9011	25
	1.8	8	11	3	0.9048	16	8	11	5	0.9030	15	8	11	3	0.9063	15	7	12	2	0.9230	13
	2.0	5	9	2	0.9046	11	5	8	4	0.9023	10	6	9	3	0.9030	10	5	8	2	0.9150	10
0.1	1.2	-	-	-	-	-	-	-	-	-	-	-	-	-	-	-	-	-	-	-	-
	1.3	66	84	1	0.9011	119	62	76	2	0.9028	106	68	82	2	0.9258	112	59	67	2	0.9050	96
	1.4	38	46	1	0.9072	71	39	64	2	0.9072	69	42	69	2	0.9125	72	37	54	1	0.9069	64
	1.5	28	39	2	0.9078	57	29	43	2	0.9147	53	27	46	2	0.9045	48	25	43	1	0.9067	45
	1.8	12	22	2	0.9087	29	13	21	2	0.9203	26	12	19	3	0.9018	23	12	18	2	0.9122	21
	2.0	8	16	1	0.9031	22	9	19	2	0.9246	20	10	14	2	0.9444	19	9	13	2	0.9296	18
0.05	1.2	-	-	-	-	-	-	-	-	-	-	-	-	-	-	-	-	-	-	-	-
	1.3	-	-	-	-	-	-	-	-	-	-	-	-	-	-	-	-	-	-	-	-
	1.4	51	69	2	0.9031	94	51	63	2	0.9036	91	48	75	2	0.9004	83	53	81	1	0.9107	82
	1.5	34	48	2	0.9002	69	36	59	2	0.9215	66	37	56	2	0.9157	63	32	56	1	0.9008	58
	1.8	17	30	2	0.9042	36	17	28	3	0.9077	34	15	25	1	0.9027	32	16	28	1	0.9223	30
	2.0	12	20	1	0.9214	28	11	18	1	0.9136	25	11	18	2	0.9120	23	12	17	2	0.9404	21

(-) represents parameters do not exist.

**Table 9:** The MDS design values for α=0.10,ν=3.1946, and a=0.50.

β	$\frac{\mu_{N}}{\mu_{0N}}$	$I_{U}=0.00$					$I_{U}=0.02$					$I_{U}=0.04$					$I_{U}=0.05$				
		$c_{1}$	$c_{2}$	m	$L\left(p_{1}\right)$	*ASN*	$c_{1}$	$c_{2}$	m	$L\left(p_{1}\right)$	*ASN*	$c_{1}$	$c_{2}$	m	$L\left(p_{1}\right)$	*ASN*	$c_{1}$	$c_{2}$	m	$L\left(p_{1}\right)$	*ASN*
0.25	1.2	13	51	2	0.9021	93	14	57	3	0.9068	91	13	41	2	0.9027	82	13	21	3	0.9031	77
	1.3	6	35	3	0.9082	47	6	42	3	0.9090	44	7	34	3	0.9291	41	6	10	3	0.9070	40
	1.4	3	7	2	0.9052	29	3	21	2	0.9089	27	4	17	3	0.9075	26	3	27	2	0.9008	25
	1.5	2	24	2	0.9080	25	2	14	3	0.9053	22	2	13	2	0.9105	21	2	13	1	0.9177	20
	1.8	1	14	6	0.9082	15	1	7	3	0.9432	14	1	12	4	0.9309	13	1	11	6	0.9206	12
	2.0	1	2	4	0.9055	15	1	6	3	0.9053	14	0	8	1	0.9049	10	14	1	6	0.9136	10
0.1	1.2	23	78	2	0.9079	168	23	38	2	0.9125	157	23	43	2	0.9093	148	22	74	2	0.9013	138
	1.3	10	37	2	0.9085	84	10	41	2	0.9059	79	10	63	2	0.9076	74	10	42	2	0.9048	72
	1.4	6	45	2	0.9224	57	6	38	2	0.9163	54	6	14	1	0.9200	53	6	26	2	0.9170	49
	1.5	4	29	2	0.9312	43	4	18	3	0.9090	40	4	24	3	0.9023	38	4	8	2	0.9238	37
	1.8	2	15	8	0.9077	28	2	17	5	0.9216	27	2	22	7	0.9069	25	2	18	5	0.9265	24
	2.0	1	14	4	0.9149	21	1	48	4	0.9216	19	1	9	3	0.9336	18	1	6	3	0.9267	16
0.05	1.2	30	78	2	0.9123	221	28	42	1	0.9005	203	31	80	2	0.9167	201	28	37	1	0.9070	183
	1.3	13	21	1	0.9111	117	14	70	2	0.9090	112	14	20	2	0.9136	104	14	44	2	0.9167	101
	1.4	8	38	2	0.9164	78	8	16	2	0.9078	74	8	36	2	0.9115	69	8	39	2	0.9194	66
	1.5	5	39	2	0.9180	56	5	17	2	0.9122	53	5	32	2	0.9085	50	5	12	2	0.9129	48
	1.8	2	6	2	0.9338	33	2	16	3	0.9081	31	2	27	3	0.9077	29	2	20	3	0.9083	28
	2.0	1	5	2	0.9166	25	1	15	2	0.9092	24	1	18	2	0.9022	23	1	19	2	0.9165	21

**Table 10:** The MDS design values for α=0.10, ν=3.1946, and a=1.00.

β	$\frac{\mu_{N}}{\mu_{0N}}$	$I_{U}=0.00$					$I_{U}=0.02$					$I_{U}=0.04$					$I_{U}=0.05$				
		$c_{1}$	$c_{2}$	m	$L\left(p_{1}\right)$	*ASN*	$c_{1}$	$c_{2}$	m	$L\left(p_{1}\right)$	*ASN*	$c_{1}$	$c_{2}$	m	$L\left(p_{1}\right)$	*ASN*	$c_{1}$	$c_{2}$	m	$L\left(p_{1}\right)$	*ASN*
0.25	1.2	20	39	2	0.9192	43	21	31	2	0.9251	40	19	29	2	0.9051	35	19	29	2	0.9153	34
	1.3	10	17	4	0.9106	21	9	12	2	0.9020	19	9	12	1	0.9113	18	10	12	3	0.9142	17
	1.4	5	14	2	0.9142	15	6	8	2	0.9027	12	5	10	2	0.9075	11	6	7	4	0.9187	10
	1.5	3	10	1	0.9088	11	4	7	2	0.9220	10	4	7	4	0.9105	9	4	6	2	0.9372	9
	1.8	1	5	2	0.9158	8	2	6	6	0.9078	6	2	5	3	0.9270	6	2	4	2	0.9400	5
	2.0	1	4	2	0.9577	6	1	3	4	0.9125	4	1	3	2	0.9374	4	2	3	16	0.9064	4
0.1	1.2	34	69	2	0.9030	70	36	45	2	0.9237	68	34	40	1	0.9064	65	35	40	2	0.9001	64
	1.3	18	36	3	0.9003	40	15	28	2	0.9096	32	16	27	1	0.9121	31	15	28	2	0.9034	30
	1.4	10	22	3	0.9093	24	9	15	2	0.9129	21	9	13	2	0.9100	20	9	13	1	0.9014	18
	1.5	7	13	2	0.9460	18	7	11	2	0.9490	17	7	15	5	0.9093	16	6	9	3	0.9007	14
	1.8	3	9	5	0.9019	10	2	5	1	0.9168	9	3	5	2	0.9491	8	3	6	2	0.9409	8
	2.0	2	7	2	0.9521	8	2	4	7	0.9151	7	2	3	6	0.9028	7	2	4	3	0.9316	7
0.05	1.2	46	85	2	0.9148	95	44	83	2	0.9050	87	44	74	2	0.9084	83	45	64	2	0.9104	81
	1.3	23	45	2	0.9185	52	22	44	2	0.9262	47	21	34	2	0.9182	43	21	36	2	0.9189	42
	1.4	12	24	2	0.9060	34	14	25	4	0.9096	32	12	21	2	0.9120	27	14	20	3	0.9191	25
	1.5	8	21	2	0.9084	22	8	16	2	0.9040	21	10	19	7	0.9082	20	7	14	2	0.9027	17
	1.8	3	12	1	0.9270	14	3	10	1	0.9038	12	3	8	2	0.9099	10	3	6	1	0.9052	10
	2.0	2	6	2	0.9203	11	2	6	1	0.9425	9	2	6	2	0.9209	8	2	3	2	0.9111	8

## Data Availability

Not applicable.

## Abbreviations

MDS	Multiple dependent state
ASN	Average sample number
SSP	Single sampling plan
COVID-19	Coronavirus disease
OC	Operating characteristic
GD	Gamma distribution
